# Unilateral absence of vas deferens: prevalence among 23,013 men seeking vasectomy

**DOI:** 10.1590/S1677-5538.IBJU.2015.0717

**Published:** 2016

**Authors:** Sarah Miller, Sophie Couture, Gareth James, Simon Plourde, Jacky Rioux, Michel Labrecque

**Affiliations:** 1 The Institute for Family Health and Montefiore Medical Center's Department of Social and Family Medicine, Albert Einstein College of Medicine, Bronx, New York, USA; 2 Research Centre of the CHU de Québec-Université Laval, Hôpital Saint-François d'Assise, Quebec City, Canada; 3 Danetre Medical Practice, Daventry, United Kingdom; 4 Clinique médicale Pierre-Bertrand, Quebec City, Canada

**Keywords:** Prevalence, Spermatic Cord, Vasectomy

## Abstract

**Purpose:**

To determine the prevalence of unilateral absence of vas deferens (UAVD) in men with both testes seeking vasectomy.

**Materials and Methods:**

Computerized charts of 23,013 patients encountered between January 1994 and December 2013 in one university hospital and two community clinics of Quebec City, Canada, were searched. Pre-vasectomy consultation, operative reports and semen analysis results were reviewed to identify cases of UAVD. Cases were categorized as confirmed (unilateral vasectomy and success confirmed by semen analysis) or possible congenital UAVD further sub-categorized according to whether or not a scrotal anomaly was present.

**Results:**

Among 159 men identified as potentially having UAVD, chart review revealed that 47 had only one testicle, 26 had bilateral vasa, and four were misdiagnosed (post-vasectomy semen analysis [PVSA] showing motile sperm after unilateral vasectomy) leaving 82 men deemed cases of UAVD (0.36%, 95% confidence interval 0.28% to 0.43%). These were classified as confirmed (n=48, 0.21%) and possible (n=34, 0.15%; 22 without and 12 with scrotal anomalies) congenital UAVD. The misdiagnosis ratio of UAVD was low when scrotal content was otherwise normal (1:48), but higher if anomalies were present (3:12).

**Conclusions:**

Most surgeons who perform vasectomy will encounter cases of UAVD. In most suspected cases, it is safe and effective to proceed with unilateral vasectomy under local anesthesia while stressing the need for PVSA. Further studies or scrotal exploration may be considered in patient with prior scrotal surgery.

## INTRODUCTION

Vasectomy is a common and safe office-based procedure. Successful sterilization by vasectomy requires interruption of both left and right vas deferens. In some men, however, bilateral procedure is not possible because of unilateral absence of the vas deferens (UAVD). UAVD may be either congenital or secondary to trauma or surgery.

For those performing vasectomies, understanding the prevalence and other clinical considerations related to unilateral absence of vas is important. Knowing how often this finding may be encountered in men seeking vasectomy can be reassuring for both experienced and less-experienced surgeons. It may prevent unnecessary surgical exploration in search of an absent vas.

Robust data on the prevalence of unilateral absence of a single vas in men seeking vasectomy, however, are lacking. Prevalence of UAVD in published reports varies widely-from 0.08% to 1.25%-with many case series not reporting the results of post-vasectomy semen analyses (PVSA) or other tests to confirm the diagnosis (1–7). The largest published case series to date includes only 12 cases of UAVD (2).

Our objective was to determine the prevalence of UAVD in a large cohort of men seeking vasectomy.

## MATERIALS AND METHODS

This retrospective descriptive study included all patients who presented seeking a vasectomy between January 1994 and December 2013 at three vasectomy centers in Quebec City, Canada: one family planning clinic located in a university hospital (2000 to 2013), and two community primary care clinics (1994 to 2013). The study was considered a medical audit, and the hospital and clinic medical directors approved data access. Because no patients were contacted and individually identifiable health information was not included in the database used for analysis, the study protocol was not submitted to an institutional review board.

### Clinical procedures

Pre-vasectomy consultation, including a genital examination, was performed on all men, usually about one month prior to vasectomy. Starting in 2009, the pre-vasectomy consultation was done by telephone in about 10% of men, most living far from the Quebec City area. Almost all vasectomies were performed by one physician (ML), or under his direct supervision in the teaching hospital. Lidocaine 2% without epinephrine was administered to provide anesthesia (8). No procedure was done under general or regional anesthesia. The no-scalpel vasectomy (NSV) approach was used to expose the vas (9).

Two different vasal occlusion techniques were performed, according to when and where the vasectomy took place. The first technique, performed until October 1999 at the community clinics, consisted of ligating the vas with two metal clips and excising about a 1cm segment of the vas between the two clips. The second, more effective technique (10–14), performed at the university hospital for all vasectomies included in the study and at the community clinics starting October 1999, consisted of thermal cautery of 1cm of the luminal mucosa of the prostatic end of the cut vas, fascial interposition over this end using a metal clip, and testicular end left open.

A PVSA was routinely recommended 8 to 12 weeks after the procedure. Standard practice at the three sites was to provide patients with instructions and semen sample container, but no reminders were sent. Men with one PVSA showing 100.000 non-motile sperm / mL or less were considered sterile and counseled to discontinue other methods of contraception (12, 15).

### Data collection

We reviewed data in the computerized database up to December 2013 in September 2014. Data had been entered by the attending physicians directly in the computerized database in the two community clinics from 1997 to 2014, and had been transcribed from paper charts by a data clerk in the community clinics from 1994 to 1996 and in hospital from 2000 to 2014.

Potential cases were identified based on whether pre-operative examination or operative comment indicated that one vas was absent, could not be palpated, or that a unilateral vasectomy was done. For these potential cases, review of operative reports was undertaken to confirm that a unilateral procedure was done and, if so, the reason for performing unilateral vasectomy. We excluded men who had a unilateral vasectomy done because one testis was absent or totally atrophic. Of course, men with only one testis can also have unilateral absence of the vas deferens; however, these men represent a very small subgroup of patients, and the presence or absence of the vas deferens on the side without a viable testis is clinically irrelevant as vasectomy on this side would be needless. We also excluded men in whom a technical problem rendered it impossible to perform the procedure on a single side despite both vasa being present.

The remaining men were considered as suspected cases of UAVD. From these, we excluded men who were found to have had a bilateral procedure reported in the surgical notes despite a suspicion of UAVD at pre-vasectomy examination. Furthermore, PVSA results for men identified as suspected cases were reviewed to confirm whether the unilateral sterilization procedure was successful. We excluded men in whom unilateral vasectomy was performed for suspected UAVD but whose PVSA showed motile sperm, suggesting that these men had bilateral vasa. The remaining men were included as cases of UAVD.

Demographic data, including number of children, age, and ethnic background, were extracted from computerized medical records. Paper chart medical records, when available, were also reviewed.

### Classification of cases

Based on research team consensus, men identified as cases of UAVD were further classified as having confirmed or possible congenital unilateral absence of vas. Our classification criteria (described below) were supported by an anonymous online survey of 36 vasectomists from the Vasectomy-Network Google group, a vasectomy discussion group composed of over 200 vasectomy providers from multiple institutions and countries.

We classified men as having a confirmed congenital UAVD if they met the following three criteria: 1) no prior surgery or trauma to genitals, 2) otherwise normal scrotal anatomy apart from the absence of one vas at the time of the pre-operative exam or procedure, and 3) confirmed sterility by PVSA after unilateral vasectomy.

All other men identified were classified as having a possible congenital UAVD. This category was further subdivided in two sub-categories based on whether scrotal anomalies were present or not because this may influence the probability of congenital UAVD. The first sub-category includes men with no prior surgery or trauma to genitals and otherwise normal scrotal anatomy who had, 1) a pre-vasectomy exam showing UAVD but who did not return for vasectomy, 2) a pre-vasectomy initial examination showing UAVD and a PVSA confirming sterility, but no surgical notes confirming vasectomy had been unilateral, or 3) surgical notes confirming that vasectomy had been unilateral because of UAVD, but who did not have a PVSA result in their medical record. The second subcategory consists in men with, 1) a single vas deferens who had undergone prior operative procedure to the testes or inguinal region, or 2) a partially atrophic testis or other scrotal anomaly on the ipsilateral side to the absent vas, as it could not be determined definitively whether the absence of the vas was associated with the surgery/scrotal anomaly or not. We did not take into account the presence or absence of ipsilateral kidney as a correlate increasing suspicion for congenital UAVD to classify men as this information was not available for most men at the time of the pre-operative exam or procedure.

## RESULTS

The computerized medical records of a total of 23,013 men were analyzed. Table-1 presents the number of procedures by location. Almost all 21.242 vasectomies were performed by one physician (ML), or under his direct supervision in the teaching hospital; 401 (1.9%) were performed by another physician between August 2002 and October 2004 and 610 (2.9%) by a co-author (SP) between November 2011 and December 2013.

**Table 1:** Number of procedures performed in the study cohort of men seeking vasectomy according to clinical settings.

Procedures	Settings		
	Community clinics (n=19.367) n (%)	Hospital (n=3.646) n (%)	Total (n=23.013) n (%)
Vasectomy	17.879 (92.3)	3.363 (92.2)	21.242 (92.3)
PVSA*	11.915 (66.6)	2.238 (66.5)	14.153 (66.6)

**PVSA**= Post-vasectomy semen analysis

* Proportion based on men who had vasectomy

**Figure 1:**
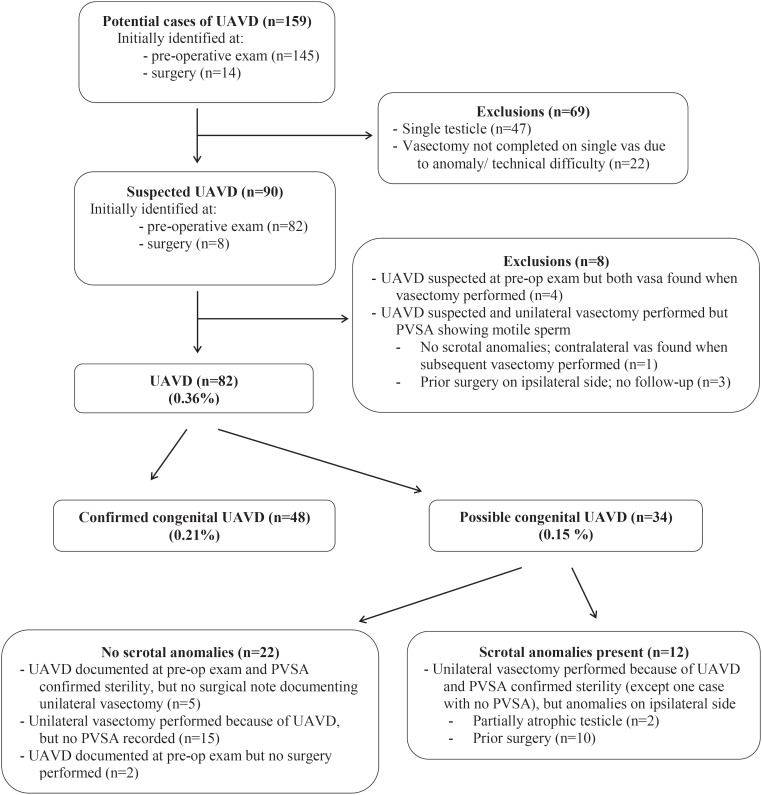
Flow chart of identification and classification of cases of unilateral absence of vas in men seeking vasectomy (N=23.013). **UAVD=** Unilateral absence of vas deferens; **PVSA**= Post-vasectomy semen analysis

Figure-1 shows the flow chart of identification and classification of cases of UAVD in men seeking vasectomy. All but four of the 159 potential cases and one of the 90 suspected cases were identified in the records of one physician (ML). Most potential and suspected cases were identified at the time of the pre-vasectomy exam.

Eighty-two (0.36%, asymptotic [Wald method (16)] 95% confidence interval [95% CI] 0.28%-0.43%) among the 23,013 men seeking vasectomy were found to have an UAVD. These were a homogenous group of Caucasians, 77 (94%) determined to be French Canadians by name. They averaged 37.1±5.4 years of age (range 26 to 52) and had a mean of 2.0±1.0 children (range 0 to 5; 9 [11.0%] men reported having never fathered children). The proportion of men with bilateral vasa reporting having never fathered children was 7.1% (1620/22926; 5 missing values). The difference was not statistically significant (Chi-square test, p=0.17).

**Figure 2:**
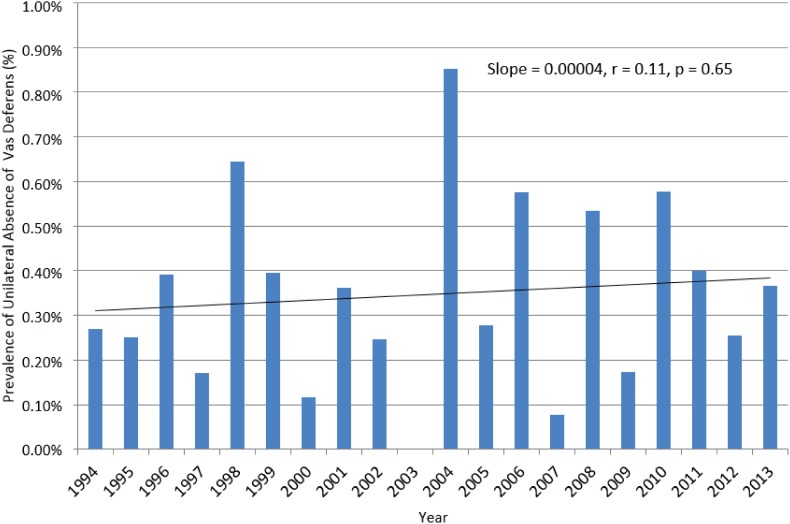
Prevalence of unilateral absence of vas deferens by year (1994-2013). **UAVD=** Unilateral absence of vas deferens; **PVSA**= Post-vasectomy semen analysis

Prevalence of UAVD was similar in the private community (0.34% [65/19.367]) and the university hospital (0.47% [17/3.646]) settings (Chi-square test, p=0.22), and by year of consultation (Figure-2).

Among the 82 UAVD cases, 48 (0.21%, 95% CI 0.15%-0.27%) were classified as confirmed congenital UAVD and the remaining 34 (0.15%, 95% CI 0.1%-0.2%) as possible cases (Figure-1). Only two men did not return for a vasectomy after pre-vasectomy initial examination showing UAVD. One of them reported having no kidney on the ipsilateral side of absent vas. Among the 80 men who had a surgery, 64 (80.0%) provided at least one semen sample for PVSA. Results for all indicated post-operative sterility. There was no spontaneous report of pregnancy after an average follow-up of 69±53 months (range 1 to 181) in the 16 men who had a unilateral vasectomy performed and who did not comply with PVSA.

## DISCUSSION

To our knowledge, this is the largest study reporting on UAVD in men seeking vasectomy. According to our results, surgeons who perform vasectomy should encounter this finding once in every 231 to 358 men seeking vasectomy (the 95% CIs of our estimate) and be able to confirm congenital UAVD once in every 374 to 668 men. Our estimate of the prevalence of UAVD in men seeking vasectomy is somewhat higher but compatible with the estimate of one case per 371 to 820 vasectomized men based on the 95% CI of the pooled results of the case series described in Table-2.

**Table 2:** Prevalence of unilateral absence of vas deferens in published case series of men seeking vasectomy.

Studies	n/N (%)
Schmidt 1966 (7)	3/432 (0.69%)
Klapproth, Young 1973 (4)	6/1000 (0.60%)
Ho 1975 (1)	1/80 (1.25%)*
Rao 1975 (6)	1/400 (0.25%)*
Bennett 1976 (3)	2/500 (0.4%)
Moss 1976 (5)	2/2500 (0.08%)
Alderman 1988 (2)	12/8879 (0.14%)*
Total	**27/13791 (0.20%; 95% CI 0.12% to 0.27%)**

**CI=** confidence interval

* Cases confirmed by post-vasectomy semen analysis (10/12 in Alderman 1988 (2))

Most physicians who perform vasectomy should expect to encounter men with UAVD in their practice. Although our study showed that the surgical intervention itself can be performed using the NSV technique in the office setting under local anesthesia, albeit unilaterally, pre-vasectomy counseling and follow-up should be specially tailored for these men.

First, counseling should include reassurance about the common nature of their anatomic variation. Second, the well-documented association between UAVD and cystic fibrosis mutation carrier status or ipsilateral renal agenesis should be considered and discussed (17–19, 20–23). Some authors have advised that further studies and evaluation be standard or is mandatory after diagnosis of unilateral vas (17–21, 23). We believe that routine renal imaging or genetic testing is not indicated in men seeking vasectomy, but should be offered in limited cases if clinically indicated.

Complete agenesis of a single vas can be found with cystic fibrosis mutations, and these conditions together are strongly associated with infertility (17, 23, 24). The assumption that the same risk for cystic fibrosis mutations and need for investigation exist in men seeking vasectomy can lead to unnecessary interventions. Men with a patent vas on the contralateral side are much less likely to have a cystic fibrosis mutation (20, 22–25). In our study about 90% of men with UAVD who seek vasectomy have fathered children, and therefore can be presumed fertile. Furthermore, men seeking vasectomy, including those who have never fathered children, do not wish to be fertile. Thus, no genetic testing needs to be offered in men with UAVD who seek vasectomy.

The co-occurrence of unilateral renal agenesis with congenital UAVD is well established (17–23, 25). Having a single kidney is however not clinically significant in the otherwise healthy man seeking surgical sterilization and does not routinely warrant further interventions other than letting the man know of the possibility that he has only one kidney. This approach is also recommended in the Faculty of Sexual & Reproductive Healthcare Clinical Guidance on Male and Female Sterilization (14).

Third, the need for and importance of PVSA to confirm sterility after unilateral vasectomy should be stressed. In this large cohort, mainly from the practice of one experienced vasectomist, suspicion of UAVD at the time of the pre-vasectomy exam was false or probably false in eight excluded suspected cases of UAVD (Figure-1). Both vasa were found at the time of the surgery in four cases. A negative PVSA after a second surgery indicates that both vasa were present in one case. In the remaining three who had had prior surgery on the ipsilateral side of suspected UAVD, the PSVAs showed at least 5 million motile sperm/mL 11 to 34 weeks after unilateral (contralateral) vasectomy, suggesting that both vasa were in fact present. The probability that the presence of motile sperm after this post-vasectomy delay is explained by recanalization is very low as vas occlusion was performed with the most effective method, mucosal cautery and fascial interposition, in all three men (10–13).

Although misdiagnosis occured in only 4 (0.017%) of the total cohort of 23,013 men, among the 82 men with UAVD it represents 4.9%, a ratio of about 1 misdiagnosis to 20 cases of UAVD. Patients should thus be informed of this increased risk of sterilization failure following unilateral vasectomy and providers should emphasize that the need for PVSA is crucial. Compliance with PVSA was appropriately higher in our vasectomized cases of UAVD than in our total population of vasectomized men (80.0% versus 66.6%).

The risk of sterilization failure appears however to vary whether men had a prior surgery or scrotal anomalies on the ipsilateral side of non-palpable vas or not. The ratio of misdiagnosis in men with otherwise normal genitals was 1 to 48 if, conservatively, only the confirmed congenital UAVD are considered as the denominator. This ratio should be similar in the 22 men with otherwise normal genitals but in whom congenital UAVD could not be fully confirmed. The risk of misdiagnosis of UAVD was much higher in men with scrotal anomalies with a ratio of 1 to 4 (3 misdiagnosis to 12 UAVD with scrotal anomalies). Referral for or proceeding to a vasectomy under general anesthesia to better evaluate the anomalous side may then be considered.

Our study has limitations. The retrospective design limited the ability to confirm or exclude congenital UAVD in all cases. However, our study design and large sample size provides a robust and precise estimate of how often the clinical suspicion of an absent vas is encountered in men seeking vasectomy. In addition, classifying our results into confirmed (59% of 82), and possible (41%) cases take into account the level of certainty of our estimates of the prevalence of congenital UAVD. The “possible” category includes cases with different probability of congenital UAVD. For example, the probability of congenital UAVD is lower in men with prior surgery on the ipsilateral side of UAVD who had negative PVSA after unilateral vasectomy than in those with no history of genital surgery in whom one vas was not found at both pre-op consultation and at the time of the surgery, even if they did not comply with the PVSA.

External validity of the study may be limited. The cohort studied represents a homogenous population of French Canadians, mostly from the practice of a single experienced physician. However, the observed prevalence is somewhat similar to previous, but much smaller, case series (1, 3, 4, 6, 7).

## CONCLUSIONS

Most surgeons who perform vasectomy will encounter a few cases of UAVD in their career and should be aware of this occurrence. When UAVD is suspected, it is safe and effective to proceed with unilateral vasectomy procedure under local anesthesia while stressing the need for PVSA to confirm sterility and informing patients about associated genetic and congenital anomalies. Further studies or scrotal exploration may be considered in patient with prior scrotal surgery.

## References

[1] (1975). Letter: Congenital absence of vas deferens. Can Med Assoc J.

[2] Alderman PM (1988). The lurking sperm. A review of failures in 8879 vasectomies performed by one physician. JAMA.

[3] Bennett AH (1976). Vasectomy without complication. Urology.

[4] Klapproth HJ, Young IS (1973). Vasectomy, vas ligation and vas occlusion. Urology.

[5] Moss WM (1976). Sutureless vasectomy, an improved technique: 1300 cases performed without failure. Fertil Steril.

[6] Rao KG (1975). Congenital absence of vas deferens. Can Med Assoc J.

[7] Schmidt SS (1966). Technics and complications of elective vasectomy. The role of spermatic granuloma in spontaneous recanalization. Fertil Steril.

[8] Shih G, Njoya M, Lessard M, Labrecque M (2010). Minimizing pain during vasectomy: the mini-needle anesthetic technique. J Urol.

[9] Li SQ, Goldstein M, Zhu J, Huber D (1991). The no-scalpel vasectomy. J Urol.

[10] Labrecque M, Dufresne C, Barone MA, St-Hilaire K (2004). Vasectomy surgical techniques: a systematic review. BMC Med.

[11] Labrecque M, Hays M, Chen-Mok M, Barone MA, Sokal D (2006). Frequency and patterns of early recanalization after vasectomy. BMC Urol.

[12] Sharlip ID, Belker AM, Honig S, Labrecque M, Marmar JL, Ross LS (2012). AUA guideline. J Urol.

[13] Sokal DC, Labrecque M (2009). Effectiveness of vasectomy techniques. Urol Clin North Am.

[14] Healthcare FoSR (2014). Male and Female Sterilisation.

[15] Dohle GR, Diemer T, Kopa Z, Krausz C, Giwercman A, Jungwirth A (2012). European Association of Urology guidelines on vasectomy. Eur Urol.

[16] Sergeant EGS (2016). AusVet Animal Health Services and Australian Biosecurity Cooperative Research Centre for Emerging Infectious Disease.

[17] Deane AM, May RE (1982). Absent vas deferens in association with renal abnormalities. Br J Urol.

[18] Donohue RE, Fauver HE (1989). Unilateral absence of the vas deferens. A useful clinical sign. JAMA.

[19] Hashimi H, Stewart AL (1991). Importance of unilateral absence of the vas deferens. Br J Surg.

[20] Kolettis PN, Sandlow JI (2002). Clinical and genetic features of patients with congenital unilateral absence of the vas deferens. Urology.

[21] Patrick JK (1982). Congenital absence of the vas deferens. Am Fam Physician.

[22] Rotman A, Hutson J (2010). Congenital unilateral absence of the vas deferens. ANZ J Surg.

[23] Weiske WH, Sälzler N, Schroeder-Printzen I, Weidner W (2000). Clinical findings in congenital absence of the vasa deferentia. Andrologia.

[24] Mickle J, Milunsky A, Amos JA, Oates RD (1995). Congenital unilateral absence of the vas deferens: a heterogeneous disorder with two distinct subpopulations based upon aetiology and mutational status of the cystic fibrosis gene. Hum Reprod.

[25] Schlegel PN, Shin D, Goldstein M (1996). Urogenital anomalies in men with congenital absence of the vas deferens. J Urol.

